# Ultrashort Peptide Hydrogels Display Antimicrobial Activity and Enhance Angiogenic Growth Factor Release by Dental Pulp Stem/Stromal Cells

**DOI:** 10.3390/ma14092237

**Published:** 2021-04-27

**Authors:** Marina E. Afami, Ikhlas El Karim, Imad About, Sophie M. Coulter, Garry Laverty, Fionnuala T. Lundy

**Affiliations:** 1 Wellcome-Wolfson Institute for Experimental Medicine, School of Medicine, Dentistry and Biomedical Sciences, Queen’s University Belfast, 97 Lisburn Road, Belfast BT9 7BL, UK; mafami01@qub.ac.uk (M.E.A.); i.elkarim@qub.ac.uk (I.E.K.); 2 Aix Marseille Univ, CNRS, ISM, Inst Movement Sci, 13385 Marseille, France; imad.about@univ-amu.fr; 3 School of Pharmacy, Queen’s University Belfast, 97 Lisburn Road, Belfast BT9 7BL, UK; Sophie.Coulter@qub.ac.uk (S.M.C.); garry.laverty@qub.ac.uk (G.L.)

**Keywords:** antibacterial, biocompatibility, dental pulp, oral pathogen, secretome

## Abstract

Recent studies on peptide hydrogels have shown that ultrashort peptides (<8 amino acids) can self-assemble into hydrogels. Ultrashort peptides can be designed to incorporate antimicrobial motifs, such as positively charged lysine residues, so that the peptides have inherent antimicrobial characteristics. Antimicrobial hydrogels represent a step change in tissue engineering and merit further investigation, particularly in applications where microbial infection could compromise healing. Herein, we studied the biocompatibility of dental pulp stem/stromal cells (DPSCs) with an ultrashort peptide hydrogel, (naphthalene-2-ly)-acetyl-diphenylalanine-dilysine-OH (NapFF*ε*K*ε*K-OH), where the epsilon (*ε*) amino group forms part of the peptide bond rather than the standard amino grouping. We tested the antimicrobial properties of NapFF*ε*K*ε*K-OH in both solution and hydrogel form against *Staphylococcus aureus*, *Enterococcus faecalis* and *Fusobacterium nucleatum* and investigated the DPSC secretome in hydrogel culture. Our results showed NapFF*ε*K*ε*K-OH hydrogels were biocompatible with DPSCs. Peptides in solution form were efficacious against biofilms of *S. aureus* and *E. faecalis*, whereas hydrogels demonstrated antimicrobial activity against *E. faecalis* and *F. nucleatum*. Using an angiogenic array we showed that DPSCs encapsulated within NapFF*ε*K*ε*K-OH hydrogels produced an angiogenic secretome. These results suggest that NapFF*ε*K*ε*K-OH hydrogels have potential to serve as novel hydrogels in tissue engineering for cell-based pulp regeneration.

## 1. Introduction

Recent progress in tissue engineering research has transformed hydrogels from their use as controlled delivery vehicles or inert cell carriers, to functional, biocompatible constructs, designed to influence the extracellular milieu in favour of tissue regeneration [1,2]. Extracellular matrix (ECM) components have been used very successfully for the development of hydrogels for tissue engineering purposes [3,4]. Indeed, new developments such as aerogels, based on natural alginate polymers are also in development for medical applications [5]. An important advantage of natural polymers, such as collagen, is its favourable biocompatibility [6]. However, variations in collagen preparation protocols (animal source and extraction methods) can lead to batch-to-batch variation in purity and yield [7], resulting in a level of unpredictability in the biochemical and/or mechanical properties of the resulting collagen hydrogel.

Self-assembling peptide hydrogels [8], represent an advanced class of hybrid (also known as smart/designer) hydrogels that are synthesised by standard peptide synthesis methods but yet retain the advantages of naturally derived polymers. The design and synthesis of bioinspired custom peptide sequences that self-assemble into hydrogels offers the possibility of tailoring the sequence of amino acids within the peptide, so that the hydrogel’s functional properties can be modified towards specific clinical applications [9]. The constituent peptides have been reported to self-assemble into nano fibrous networks to form hydrogels on the basis of the amphipathicity and/or self-complementarity of their peptide sequences [10]. Self-assembly is also facilitated by hydrophobic stacking interactions, generally in the form of *π*–*π* interactions, in the presence of aromatic amino acids (e.g., phenylalanine) or bulky groups such as 9-fluorenylmethyloxycarbonyl (Fmoc) [11,12]. In general, relatively short peptide sequences (8–25 amino acids), have been employed for hydrogel synthesis [13] and in addition to retaining many of the favourable characteristics of naturally-derived proteins, they are relatively inexpensive to synthesise. Ultrashort peptides (<8 amino acids) have also been shown to self-assemble into hydrogels [14], and have the added advantage of even lower cost synthesis with increased translationary potential.

Interestingly, within the class of self-assembling ultrashort peptides, it has been possible to design antimicrobial motifs, particularly those incorporating positively charged lysine residues, such that the peptides have inherent antimicrobial characteristics [15,16]. The design and synthesis of biocompatible hydrogels with antimicrobial activity is of great interest for tissue engineering purposes involving replacement of tissue in infected root canals [17,18,19]. It is well recognised that successful cell-based regenerative endodontics, involving transplanted cells, requires a high level of disinfection [20]. Indeed, it has been shown experimentally that residual bacteria have a critical negative effect on the success of regenerative endodontic procedures [21]. Disinfection of the root canals generally involves the use of irrigants, such as sodium hypochlorite and intracanal medicaments such as antibiotics. However, these approaches are not without their limitations and concerns have been raised that sodium hypochlorite may interfere with the ability of the regenerating pulp tissue to reattach to the dentin surface [22] and there is an urgent need to reduce antibiotic usage [23].

The dental pulp contains multipotent mesenchymal progenitor cells, known as dental pulp stem/stromal cells (DPSCs), which participate in dentin and pulp regeneration [24] and have been used in tissue engineering. To date, antimicrobial hydrogels have not been studied extensively for their biocompatibility with DPSCs or for their ability to inhibit micro-organisms (particularly those in biofilm form) relevant to tissue engineering sites such as root canals. Herein we assessed the biocompatibility of DPSCs with an ultrashort self-assembling peptide, (naphthalene-2-ly)-acetyl-diphenylalanine-dilysine-OH (NapFF*ε*K*ε*K-OH) whereby the epsilon (*ε*) amino group forms part of the peptide bond rather than the standard amino grouping [16]. We investigated the antimicrobial activity of NapFF*ε*K*ε*K-OH against *Staphylococcus aureus*, *Enterococcus faecalis* and *Fusobacterium nucleatum* and determined effects on angiogenic growth factor expression by DPSCs in NapFF*ε*K*ε*K-OH hydrogel culture.

## 2. Materials and Methods

### 2.1. Peptide Synthesis and Gelation

NapFF*ε*K*ε*K-OH was synthesised on a manual nitrogen bubbler apparatus using the 9-fluorenylmethoxucarbonyl (Fmoc) solid phase peptide synthesis chemistry as previously outlined [16]. Hydrogel formulation was achieved by a pH triggered method as previously described [16,25] and outlined in further detail in Table 1. Depending on the peptide concentration required (0.5%–2%; measured as % weight/volume (*w*/*v*)), the required amount of peptide was dissolved in an appropriate volume of deionised water (dH_2_O) and titrated with 1 M sodium hydroxide (NaOH) to pH 8.5 and 0.5 M hydrochloric acid (HCl) to pH 7. Assembled hydrogels were examined by gel inversion assay to determine if they were self-supporting [16]. Hydrogels were employed in modified 3-(4,5-dimethylthiazol-2-yl)-2,5-diphenyltetrazolium bromide (MTT) assays, encapsulation studies, live/dead assays, secretome studies and bacterial susceptibility assays. The antibiofilm activities of NapFF*ε*K*ε*K-OH peptides in solution were also investigated. For antibiofilm studies, NapFF*ε*K*ε*K-OH peptides were retained in solution by dissolving in alpha minimum essential medium (*α*-MEM) containing 0.005% *v*/*v* dimethyl sulphoxide (DMSO), as an alternative to pH triggered hydrogel formation.

### 2.2. Dental Pulp Cell Culture

Dental pulp samples [26] were obtained in accordance with French ethics legislation and covered by the Office for Research Ethics Committees (Northern Ireland) ethical approval number 08/NIR03/15. Dental pulp tissue was cut into fragments and cultured using the explant method [27]. DPSCs were cultured in *α*-MEM (Thermo Fisher Scientific, Waltham, MA, USA), supplemented with 10% heat inactivated foetal bovine serum (Thermo Fisher Scientific, Waltham, MA USA), 1% *w*/*v* L-glutamine (L-glut) (Thermo Fisher Scientific, Waltham, MA, USA) and 1% *w*/*v* penicillin + streptomycin (Thermo Fisher Scientific, Waltham, MA, USA). Cells were grown to approximately 80% confluence at 37 °C with 5% CO_2_. Cells between passages 2–4 were used throughout.

### 2.3. Modified MTT Assay for DPSCs in 3D Culture

DPSCs (1 × 10^6^ cells/mL) were encapsulated in 0.5% to 2% *w*/*v* NapFF*ε*K*ε*K-OH hydrogels (100 µL) and 200 µL of *α*-MEM gently added on top to establish 3D culture in 96-well plates. Viability of DPSCs encapsulated within NapFF*ε*K*ε*K-OH hydrogels was assessed by employing an MTT protocol, previously modified for 3D cell cultures [28]. DPSCs encapsulated in 2% *w*/*v* hydroxypropyl methylcellulose (HPMC) hydrogels were included as 3D controls. Cell viability was assessed at 1 and 14 days after encapsulation. Briefly, 20 µL of MTT (5 mg/mL in Hanks balanced salt solution (HBSS)) was added to each well and incubated for 1 h at 37 °C. Media (200 µL) was then carefully removed and replaced by 200 µL DMSO with incubation for 10 min at 37 °C. The converted dye was eluted by shaking the plate at 220 rpm for 3 h. The supernatant from each well was transferred to a new plate and the absorbance measured at 570 nm on a microplate reader (Thermo Scientific Varioskan LUX, Warrington, UK) using SkanIt RE 4.1 software.

### 2.4. Visualisation of Encapsulated Cells

To visualise cell encapsulation within hydrogels by confocal microscopy, DPSCs were encapsulated in 0.5–2% *w*/*v* NapFF*ε*K*ε*K-OH hydrogels at a density of 1 × 10^6^ cells/mL, with 100 µL of cells encapsulated in 100 µL of hydrogel. Prior to plating the encapsulated cells in glass bottom dishes (WillCo Wells BV, Amsterdam, The Netherlands) the bottom of each dish was marked with a hydrophobic barrier pen (Fisher Scientific, Dublin, Ireland), to retain the hydrogels within a defined area. Encapsulated DPSCs were incubated for 24 h before fixing with 4% paraformaldehyde for 30 min and then washed with phosphate buffered saline (PBS) three times. A few drops of ProLongTMGold Antifade Mountant with 4′,6-diamidino-2-phenylindole (DAPI; ThermoFisher Scientific, Warrington, UK) were transferred to a circular coverslip, placed on top of the hydrogel construct and cured at room temperature for 24 h. Encapsulated DPSCs were visualised with SP8 Confocal Microscope (Leica). Z stack images were acquired and processed using LAS X software (Leica Microsystems, Wetzlar, Germany).

### 2.5. Live/Dead Assay

More extensive cytotoxicity studies were undertaken on 0.5% *w*/*v* NapFF*ε*K*ε*K-OH hydrogels as this was the previously reported minimum gelation concentration [16] of the hydrogel and would have favourable injectability for use in root canals. Conditioned media collected on day 14 from 0.5% *w*/*v* NapFF*ε*K*ε*K-OH hydrogels was tested for potential cytotoxic effects on DPSCs. Cells (1 × 10^4^ cells/mL) were plated in 96-well microtitre plates and incubated for 48 h. DPSCs were then treated with 100 µL of conditioned media for 24 h. Controls were treated with 5% *v*/*v* Triton-X or media. Cell viability was assessed using the live/dead viability/cytotoxicity kit, for mammalian cells (ThermoFisher Scientific, Warrington, UK) as outlined in the manufacturer’s instructions. Images were captured using an Evos FL Auto Imaging System (ThermoFisher Scientific, Warrington, UK).

### 2.6. Antibacterial Effects

To gain insight into the antibacterial action of NapFF*ε*K*ε*K-OH, we tested its efficacy in both solution and hydrogel form. The antibacterial effects of low concentrations of NapFF*ε*K*ε*K-OH peptides in solution (representing peptides in solution that may diffuse from the hydrogel surface [15,16]) were tested against bacterial biofilms in 96-well microtitre plates. *S. aureus* (ATCC 25923) and *E. faecalis* (NCTC 12697) were grown aerobically in Müeller Hinton broth, allowed to reach mid-Log phase and then diluted to 1 × 10^6^ colony forming units (CFU)/mL in Müeller Hinton broth (Sigma-Aldrich, Dorset, UK) to obtain inoculums for the biofilm assay. Wells containing 100 µL of inoculum were incubated aerobically for 5 h to establish initial *S. aureus* and *E. faecalis* biofilms. *F. nucleatum* (NCTC 10562) was grown anaerobically in fastidious anaerobe broth (LabM Limited, Lancashire, UK), allowed to reach mid-Log phase and diluted to 5 × 10^6^ CFU/mL in fastidious anaerobe broth to obtain an inoculum for the biofilm assay. Wells containing 100 µL of inoculum were incubated anaerobically for 48 h to establish initial *F. nucleatum* biofilms.

After initial biofilm formation, planktonic bacteria were removed by washing with PBS and the biofilms were treated with 100 µL of 0.01% or 0.1% *w*/*v* NapFF*ε*K*ε*K-OH (dissolved in appropriate broth containing 0.005% *v*/*v* DMSO). Following NapFF*ε*K*ε*K-OH treatment, biofilms were allowed to mature either aerobically for a further 24 h (*S. aureus* and *E. faecalis*) or anaerobically for a further 48 h (*F. nucleatum*). Wells containing inoculum were also treated with vehicle controls (broth containing 0.005% *v*/*v* DMSO). After either 24 or 48 h incubation, planktonic bacteria were removed by washing and the biofilm biomass was quantified by the crystal violet assay [29].

The ability of the 0.5% *w*/*v* assembled hydrogel to reduce bacterial viability was assessed using a colony count method as previously described [30]. Briefly, *S. aureus* and *E. faecalis* were grown aerobically in Müeller Hinton broth, allowed to reach mid-Log phase and diluted to 2 × 10^6^ CFU/mL. *F. nucleatum* was grown anaerobically in fastidious anaerobe broth, allowed to reach mid-Log phase and diluted to 5 × 10^6^ CFU/mL. Bacterial suspensions (100 µL) were added on top of 100 µL preformed 0.5% *w*/*v* NapFF*ε*K*ε*K-OH hydrogels in 96-well plates. Untreated control wells contained inoculated broth only. After 24 h incubation 20 µL samples were removed from each well, serially diluted and 10 µL from each dilution was plated out for viability counting using the Miles and Misra method [31]. Results were displayed as mean Log_10_ CFU/mL.

### 2.7. Angiogenic Array

Angiogenic arrays (human angiogenesis antibody array-membrane; Abcam Cambridge UK) were employed for the simultaneous detection of 43 growth factors and cytokines in DPSCs cultured in 2D and 3D. The assay procedure was performed as described in the manufacturer’s instructions, using 14 day conditioned medium from DPSCs cultured in either 0.5% *w*/*v* NapFF*ε*K*ε*K-OH hydrogels or standard 2D culture. Arrays were imaged using a Syngene G: BOX, with GeneSys software and semiquantified using ImageJ, image analysis programme, with a microarray plugin. Results were presented as a heat map generated using Microsoft Excel.

### 2.8. Statistical Analysis

Results were expressed as the mean of three independent experiments with three to six replicates per experiment. GraphPad Prism Software (Version 8) was used to perform statistical analysis (GraphPad Software Inc., San Diego, CA, USA). MTT assay results were analysed by one-way ANOVA followed by Dunnett’s post hoc multiple comparison test. Comparisons were made with HPMC controls in the modified MTT assay. Biofilm inhibition and bacterial viability results were analysed by Mann–Whitney non parametric tests, and comparisons were made with untreated controls or vehicle control treatments respectively. The level of statistical significance was set at *p* < 0.05.

## 3. Results

### 3.1. Peptide Hydrogels and Their Biocompatibility for 3D Culture of DPSCs

**Figure 1:**
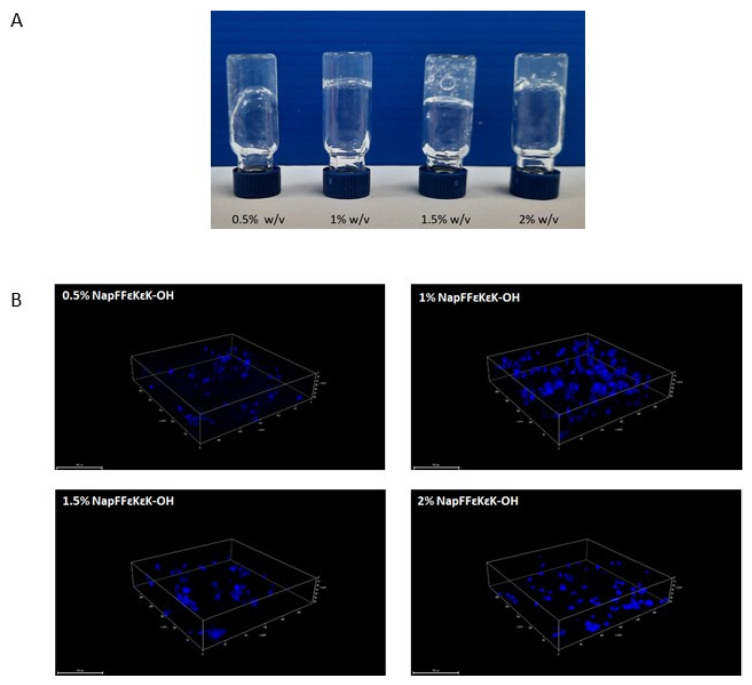
Formulated NapFF*ε*K*ε*K-OH hydrogel properties. (**A**) The assembled NapFF*ε*K*ε*K-OH hydrogels (0.5–2% *w*/*v*) were shown to be self-supporting. (**B**) Confocal microscopy of DPSCs 24 h after their encapsulation in 0.5–2% *w*/*v* NapFF*ε*K*ε*K-OH hydrogels, showed that cells were retained in 3D within the hydrogels. Cells were fixed and permeabilised within the hydrogel before staining of the encapsulated DPSC nuclei with DAPI. Images were captured using an SP8 Confocal Microscope. Scale bar: 100 µm.

**Figure 2:**
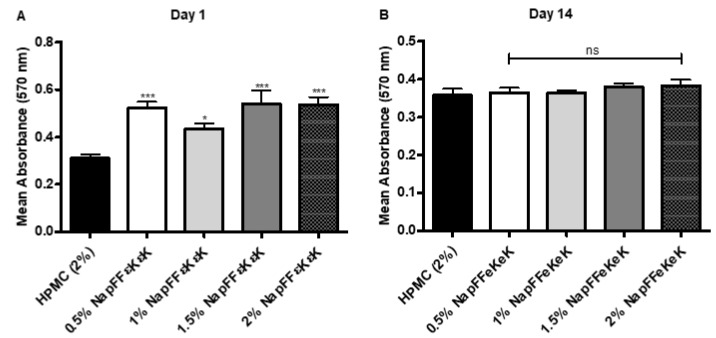
Modified MTT assay (for 3D culture) following encapsulation of DPSCs in NapFF*ε*K*ε*K-OH hydrogels (0.5%, 1%, 1.5% and 2% *w*/*v*) at (**A**) day 1 and (**B**) day 14. Results are the average (±standard error) of three independent experiments (three replicates per experiment). All comparisons were made with the HPMC control. One-way ANOVA followed by Dunnett’s post hoc correction for multiple comparisons. ns: no significant difference (*p* > 0.05), *: *p ≤* 0.05, ***: *p ≤* 0.001.

Assembled NapFF*ε*K*ε*K-OH peptide hydrogels (0.5–2% *w*/*v*) were shown to be self-supporting as determined by gel inversion assay (Figure 1A). Following their encapsulation into hydrogels, cells were fixed and permeabilised prior to DAPI staining and visualisation by confocal microscopy. Confocal images (Figure 1B) confirmed that DPSCs were maintained in 3D within 0.5–2% *w*/*v* hydrogels, following their encapsulation. Using a modified MTT assay, we tested the suitability of NapFF*ε*K*ε*K-OH hydrogels (0.5–2% *w*/*v*) for 3D culture of DPSCs after 1 and 14 days encapsulation. MTT assay results were significantly higher in all NapFF*ε*K*ε*K-OH hydrogels relative to the control HPMC hydrogel on day 1 following encapsulation (Figure 2A), whereas no significant differences were observed after 14 days (Figure 2B).

### 3.2. Live/Dead Assay of DPSCs Incubated with 0.5% w/v NapFFεKεK-OH Conditioned Medium

**Figure 3:**
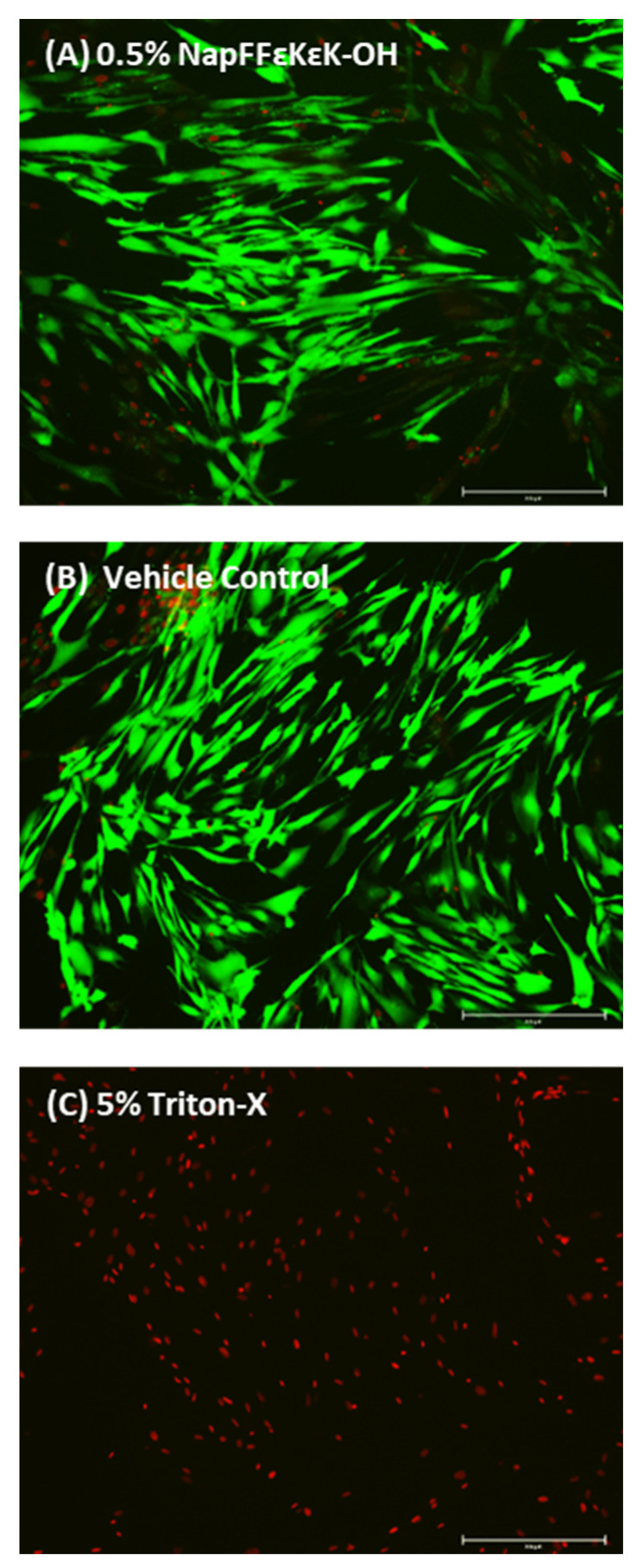
Live/dead viability/cytotoxicity assay showing representative images of (**A**) DPSCs treated with Day 14 conditioned media obtained from 0.5% *w*/*v* NapFF*ε*K*ε*K-OH hydrogels, (**B**) vehicle control (*α*-MEM incubated for 14 days at 37 °C prior to cell treatment) and (**C**) DPSCs treated with 5% *v*/*v* Triton-X. Images A and B show mainly live cells, stained green with calcein AM, with very few cells stained red, whereas in C dead cells are stained red with EthD-1. Images were captured using an Evos FL Auto Imaging System. Scale bars: 250 µm.

Further studies were undertaken using conditioned media from 0.5% *w*/*v* NapFF*ε*K*ε*K-OH hydrogels, as it represented the minimum gelation concentration [16]. DPSCs treated with 0.5% *w*/*v* NapFF*ε*K*ε*K-OH conditioned media, stained green with calcein AM, indicating live cells. Very few cells stained red with EthD-1 (dead cells), suggesting little or no cytotoxicity of soluble hydrogel components present in the conditioned medium after 14 days (Figure 3).

### 3.3. Antibacterial Activity of NapFFεKεK-OH

**Figure 4:**
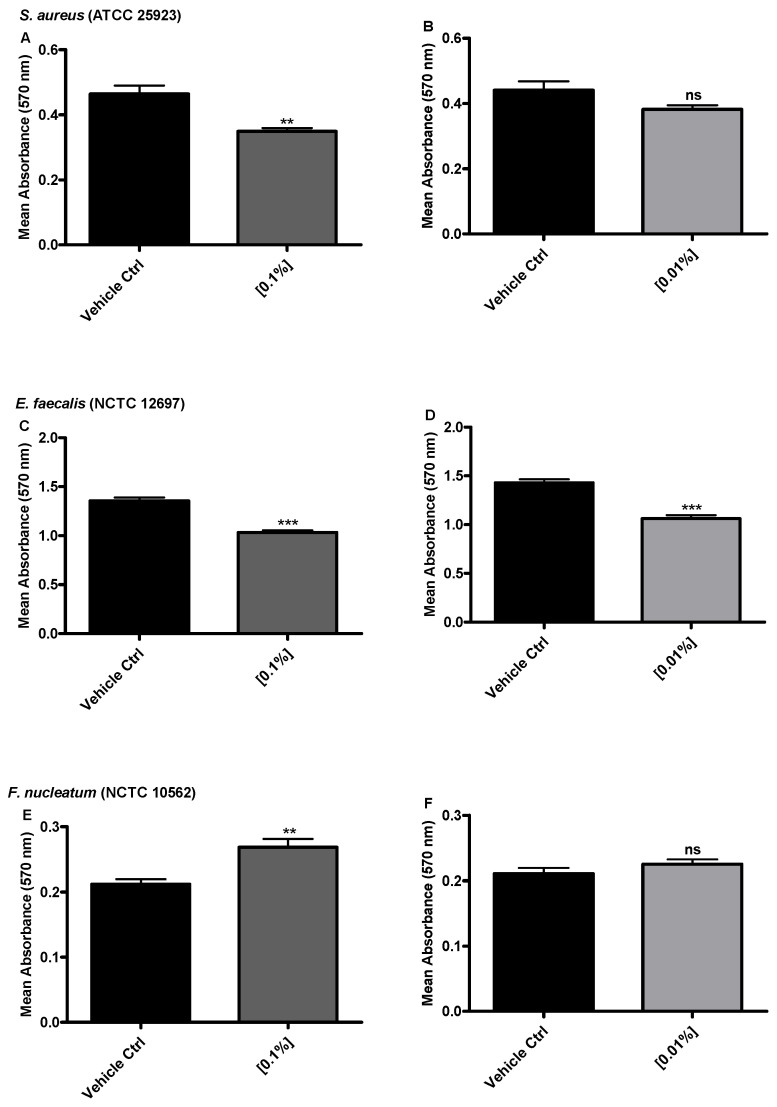
Biofilm inhibition determined by biofilm biomass using the crystal violet assay. (**A**,**B**) *S. aureus,* (**C**,**D**) *E. faecalis* and (**E**,**F**) *F. nucleatum* biofilm biomass after 24 h incubation with 0.1% and 0.01% *w*/*v* peptide concentration of NapFF*ε*K*ε*K-OH. Results are displayed as the average (±standard error) of three independent experiments (three replicates per experiment). All comparisons were made with the appropriate vehicle control. Mann–Whitney non parametric testing was employed for statistical analysis. ns: no significant difference (*p* > 0.05), **: *p ≤* 0.01, ***: *p ≤* 0.001.

It was important to determine if low concentrations (0.1%, 0.01% *w*/*v*) of NapFF*ε*K*ε*K-OH peptides (representing peptides in solution that may diffuse from the hydrogel surface) could have antimicrobial activity against biofilms of oral pathogens. Results showed that the biomass of *S. aureus* biofilms was significantly reduced when exposed to 0.1% *w*/*v* NapFF*ε*K*ε*K-OH, whereas treatment with 0.01% *w*/*v* peptide did not significantly alter biofilm biomass (Figure 4A,B). NapFF*ε*K*ε*K-OH caused a significant reduction in the biomass of *E. faecalis* biofilm at both concentrations tested (Figure 4C,D), however no reductions were observed against *F. nucleatum* biofilms (Figure 4E,F).

**Figure 5:**
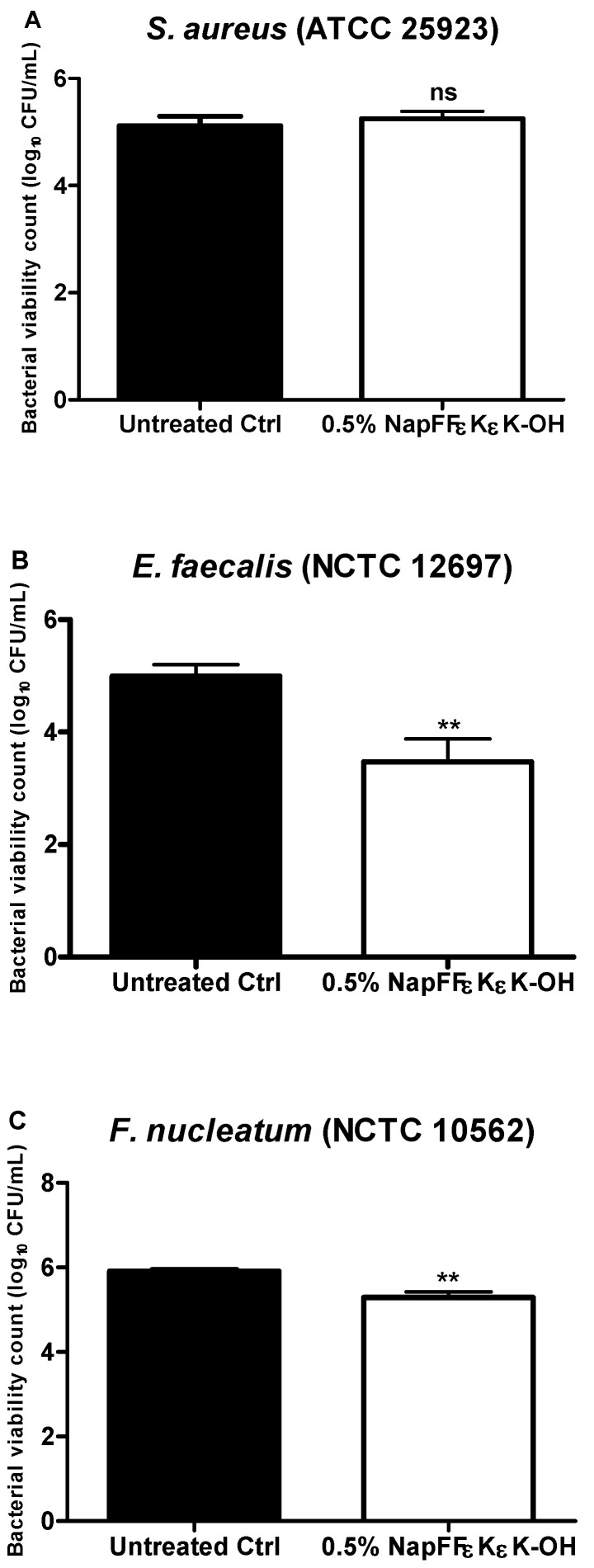
Bacterial viability counts (Log10 CFU/mL) for (**A**) *S. aureus*, (**B**) *E. faecalis* and (**C**) *F. nucleatum* after 24 h growth on the surface of 0.5% *w*/*v* NapFF*ε*K*ε*K-OH hydrogels. Results are the average (±standard error) of three independent experiments (three replicates per experiment). All comparisons were made with untreated controls. Mann–Whitney non parametric testing was employed for statistical analysis. ns: no significant difference (*p* > 0.05), **: *p ≤* 0.01.

The antibacterial activity of the assembled 0.5% *w*/*v* NapFF*ε*K*ε*K-OH hydrogel against *S. aureus*, *E. faecalis* and *F. nucleatum* was tested using a CFU viable count assay, after 24 h bacterial growth on the hydrogel surface. There was no significant change in the number of viable colonies observed when *S. aureus* was grown on the surface of NapFF*ε*K*ε*K-OH (Figure 5A). However, the NapFF*ε*K*ε*K-OH hydrogel significantly reduced numbers of *E. faecalis* (Figure 5B) and *F. nucleatum* (Figure 5C), indicating that the hydrogel itself also had significant antimicrobial activity against these micro-organisms.

### 3.4. Effect of Hydrogel Culture on the Angiogenic Secretome of DPSCs

**Figure 6:**
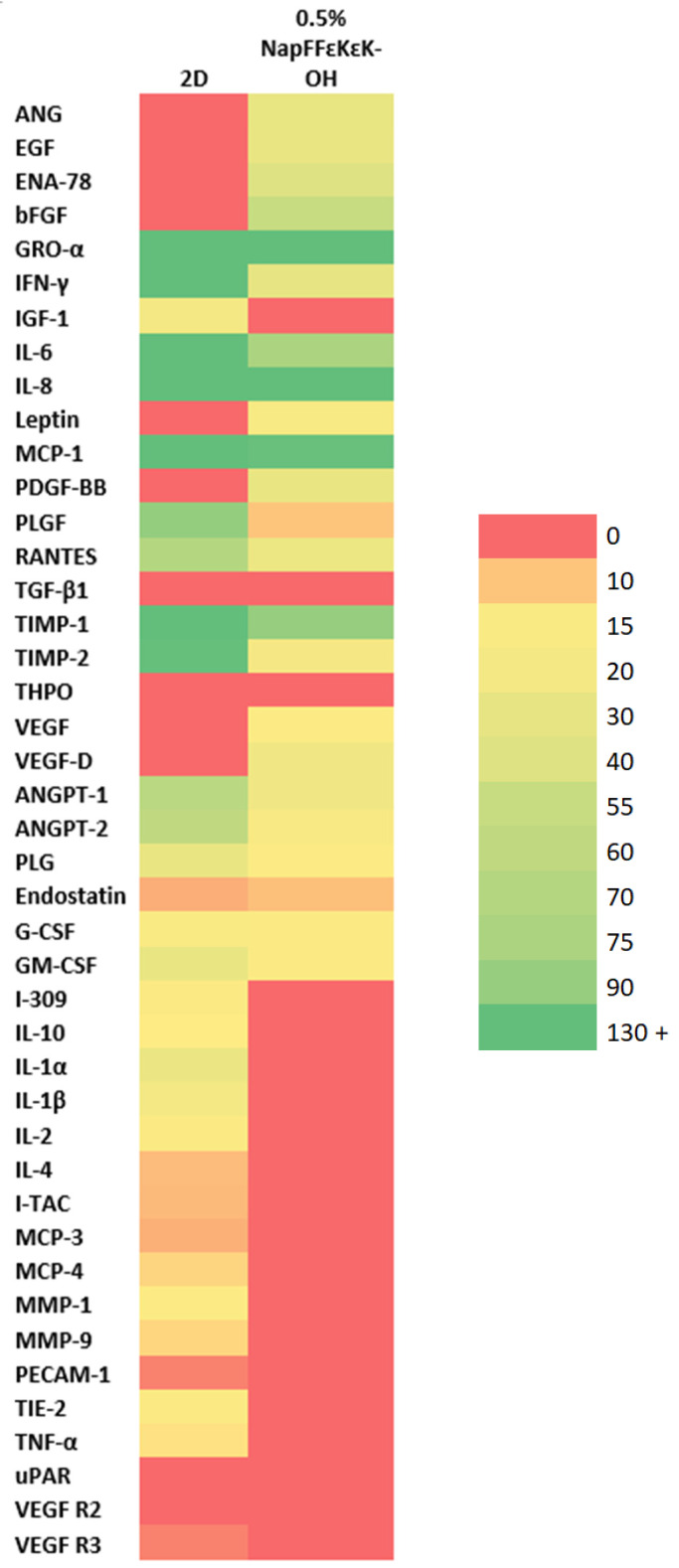
Heat map showing the expression of growth factors/cytokines in conditioned media from DPSCs following 14 days encapsulation in 0.5% *w*/*v* NapFF*ε*K*ε*K-OH hydrogels versus 2D culture. Heat map comparison showed upregulation of ANG, EGF, ENA-78, bFGF, leptin, PDGR-BB, VEGF and VEGF-D, along with downregulation of IFN-*γ*, IGF-1, IL-6, PLGF, RANTES, TIMP-1, TIMP-2, ANGPT1, ANGPT2, I-309, IL-10, IL-1*α*, IL-1*β*, IL-2, IL-4, I-TAC, MCP-3, MCP-4, MMP-1, MMP-9, TIE-2 and TNF*α* in 0.5% *w*/*v* NapFF*ε*K*ε*K-OH 3D hydrogel, compared with 2D culture. The heat map was generated in Excel using a 3-colour scale whereby red colour represents no expression above baseline, yellow represents the median expression level of the dataset and green represents the 90% percentile of the expression levels obtained. Colour shading and associated arbitrary values were then generated automatically by Excel. Results represent pooled conditioned media collected from six replicates. Abbreviations: angiogenin (ANG); epidermal growth factor (EGF); epithelial neutrophil-activating peptide 78 (ENA-78); basic fibroblast growth factor (bFGF); growth-regulated alpha protein (GRO-*α*); interferon gamma (IFN-*γ*); insulin-like growth factor 1 (IGF-1); interleukin-6 (IL-6); interleukin-8 (IL-8); leptin; monocyte chemoattractant protein-1 (MCP-1); platelet-derived growth factor BB (PGDF-BB); placental growth factor (PLGF); regulated upon activation normal T cell expressed and secreted (RANTES); transforming growth factor-beta 1 (TGF-*β*1); tissue inhibitor of matrix metalloproteinases-1 (TIMP-1); tissue inhibitor of matrix metalloproteinases-2 (TIMP-2); thrombopoietin (THPO); vascular endothelial growth factor (VEGF); vascular endothelial growth factor-D (VEGF-D); angiopoietin-1 (ANGPT1); angiopoietin-2 (ANGPT2); plasminogen (PLG); endostatin; granulocyte colony stimulating factor (G-CSF); granulocyte-macrophage colony-stimulating factor (GM-CSF); T lymphocyte-secreted protein I-309 (I-309); interleukin-10 (IL-10); interleukin-1 alpha (IL-1*α*); interleukin-1 beta (IL-1*β*); interleukin-2 (IL-2); interleukin-4 (IL-4); interferon-inducible T cell alpha chemoattractant (I-TAC); monocyte chemoattractant protein-3 (MCP-3); monocyte chemoattractant protein-4 (MCP-4); matrix metalloproteinase-1 (MMP-1); matrix metalloproteinase-9 (MMP-9); platelet and endothelial cell adhesion molecule-1 (PECAM-1); tyrosine kinase with immunoglobulin-like and EGF-like domains (TIE-2); tumour necrosis factor alpha (TNF*α*); urokinase plasminogen activator surface receptor (uPAR); vascular endothelial growth factor receptor 2 (VEGF R2); vascular endothelial growth factor receptor 3 (VEGF R3).

An angiogenic array for the detection of 43 growth factors/cytokines was employed to test conditioned media collected from DPSCs following 14 days culture in 0.5% *w*/*v* NapFF*ε*K*ε*K-OH hydrogels compared with cells grown in standard 2D DPSC culture. Heat map comparison of DPSCs grown in 0.5% *w*/*v* NapFF*ε*K*ε*K-OH 3D hydrogel compared with 2D culture, showed upregulation of angiogenin (ANG), epidermal growth factor (EGF), epithelial neutrophil-activating peptide 78 (ENA-78), basic fibroblast growth factor (bFGF), leptin, platelet-derived growth factor-BB (PDGR-BB), vascular endothelial growth factor (VEGF) and VEGF-D, along with downregulation of interferon gamma (IFN-*γ*), insulin-like growth factor 1 (IGF-1), interleukin (IL)-6, placental growth factor (PLGF), regulated upon activation normal T cell expressed and secreted (RANTES), tissue inhibitor of matrix metalloproteinases (TIMP)-1, TIMP-2, angiopoietin-1 (ANGPT1), angiopoietin-2 (ANGPT2), T lymphocyte-secreted protein I-309 (I-309), IL-10, IL-1*α*, IL-1*β*, IL-2, IL-4, interferon-inducible T cell alpha chemoattractant (I-TAC), monocyte chemoattractant protein (MCP)-3, MCP-4, MMP (matrix metalloproteinase)-1, MMP-9, tyrosine kinase with immunoglobulin-like and EGF-like domains (TIE-2) and tumour necrosis factor alpha (TNF*α*) (Figure 6).

## 4. Discussion

In the current study, NapFF*ε*K*ε*K-OH was studied at gelation concentrations (0.5–2% *w*/*v*) and biocompatibility with DPSCs was demonstrated at all concentrations tested. Previous work demonstrated that NapFF*ε*K*ε*K-OH had a nanofibrous hydrogel structure, as determined by scanning electron microscopy [16]. We selected 0.5% *w*/*v*, for further investigation as this was the minimum gelation concentration previously reported for NapFF*ε*K*ε*K-OH to form the required hydrogel platform [16]. Such low molecular peptide hydrogelator systems form shear thinning soft gels, flowing readily upon injection and recovering their viscosity quickly after removal of shear [32]. In line with previous reports on peptides containing Fmoc-FF and Nap-FF [33,34], we proposed similar rheological properties for NapFF*ε*K*ε*K-OH. Such properties would be considered advantageous in terms of ease of handling, especially for clinical applications such as injection into root canals.

The current study builds on our previous work in which a number of ultrashort peptide hydrogels were designed and tested for their antimicrobial activities [16]. Previously we showed that NapFFKK was more antimicrobial than NapFF*ε*K*ε*K. However, we also reported that NapFFKK was particularly cytotoxic to fibroblast cell lines and that NapFF*ε*K*ε*K possessed the greatest cytocompatibility of all the peptides studied [16]. Indeed, in preliminary investigations we confirmed, as expected, that NapFFKK was toxic to DPSCs (results not shown) and it was not investigated further. Instead, we focused on investigating the biocompatibility of NapFF*ε*K*ε*K-OH against DPSCs, encapsulated within the NapFF*ε*K*ε*K-OH hydrogels, using a modified MTT assay [28]. In previous reports, biocompatibility and cell growth have been investigated on hydrogel surfaces [35,36], however, the modified MTT assay [28] has the advantage that it determines biocompatibility of cells within the hydrogel and more closely represents the cell-based therapeutic approach that would be adopted for delivery of DPSCs into root canals. Using conditioned medium obtained from 0.5% *w*/*v* NapFF*ε*K*ε*K-OH hydrogels, we showed that soluble components from the hydrogel did not exhibit cytotoxic effects as determined by the live/dead assay. This assay has been extensively employed for discriminating live and dead cells in various culture conditions or following cell treatments [28,36,37].

Peptide hydrogels have favourable characteristics such as chemical and functional versatility however one potential disadvantage is their cost of synthesis and purification of larger peptide/protein motifs. NapFF*ε*K*ε*K-OH has a major advantage in that it is an ultrashort peptide and therefore relatively inexpensive to synthesise and scale-up for pharmaceutical use. Furthermore, like the majority of naturally occurring antimicrobial peptides (AMPs) [38], NapFF*ε*K*ε*K-OH is cationic. Cationic peptides, with excess numbers of positively charged amino acids (such as lysine, arginine and histidine), tend to exhibit antimicrobial activity as a result of their electrostatic interactions with negatively charged bacterial membranes [39]. Oral bacteria growing in biofilms have been reported to be more resistant to antimicrobial agents such as amoxicillin, metronidazole and doxycycline than planktonic cells [40,41] and additional therapeutics are therefore needed. NapFF*ε*K*ε*K-OH peptides in solution form (0.01% and 0.1% *w*/*v*) were tested for their antibiofilm properties to investigate if low concentrations of peptides in solution possess antibiofilm effects. It has previously been debated that antimicrobial activity is not exclusive to the process of gelation and that the presence of soluble peptides around the hydrogel surface could also have a role in the antimicrobial properties of the peptide [15,16]. The presence of lysine, and the cationic activity associated with it, undoubtedly contribute to the antibacterial activity observed in the current study. The peptides in solution are likely to act similarly to cationic AMPs, by targeting the bacterial membrane [39]. Furthermore, the antibiofilm activity of an AMP is not only related to its ability to interact with bacterial membrane but on its ability to penetrate the biofilm matrix [42]. NapFF*ε*K*ε*K-OH significantly reduced the biofilm formed by *S. aureus* only at 0.1% *w*/*v*, but it was able to significantly reduce biofilms formed by *E. faecalis* at both concentrations tested suggesting that NapFF*ε*K*ε*K-OH may exhibit different killing effects against different types of Gram positive bacteria. Moreover, no antibiofilm activity was evident against *F. nucleatum*. The structure of the bacterial cell wall differs fundamentally in Gram positive and Gram negative bacteria [43]. Whereas Gram positive bacteria have a thick peptidoglycan layer, Gram negative bacteria have a thinner peptidoglycan layer and an additional outer membrane containing lipopolysaccharide. The additional protection offered by the outer membrane [43] is thought to contribute to the tendency for Gram negative bacteria tend to be more resistant to antimicrobials (particularly in biofilm form), concurring with the results presented herein.

The use of different assays for determining antibacterial efficacy is also worth noting, as our results demonstrated both antibiofilm and bactericidal activity for NapFF*ε*K*ε*K-OH in solution and hydrogel form respectively. Much less is known about the potential mechanism of action of assembled peptides in the hydrogel form, but peptide self-assembly has been suggested to have a role in the antimicrobial properties of peptide hydrogels [44]. Previous work has also suggested that polymer-derived hydrogels may act via a contact-dependent involving membrane disruption [45]. Thus, presence or absence of gelation could contribute to the differences we observed in the efficacy of the hydrogel and solution forms of NapFF*ε*K*ε*K-OH against the same bacterial species. Moreover, the fact that a bacterial susceptibility assay was employed for determining antimicrobial activity of hydrogels whereas a biofilm assay was employed for determining the antimicrobial activity of peptides in solution, makes comparisons of peptide efficacy within the same species more challenging.

The NapFF*ε*K*ε*K-OH hydrogel, with its efficacy against relevant pathogenic bacteria, could contribute to restoring antimicrobial function at the initial stages of tissue regeneration in cell based regenerative endodontics. Moreover, the cells within the hydrogel are also likely to contribute antibacterial potential [46] during the dental pulp regenerative process. For example, dental pulp fibroblasts produce complement C3b for opsonisation of cariogenic bacteria [47] and also produce a membrane attack complex, which leads to pathogen lysis by osmotic shock [48].

One of the proposed benefits of growth of cells in 3D culture is that the cellular microenvironment is more physiologically relevant for tissue engineering purposes [49]. Therefore, hypothesising that hydrogel culture could offer a more favourable microenvironment for DPSCs, we assessed the angiogenic secretome of DPSCs grown in NapFF*ε*K*ε*K-OH compared with standard 2D culture. Angiogenesis, the sprouting of new blood vessels, plays a significant role in successful tissue engineering. So-called reparative angiogenesis, which occurs post injury or infection, recreates functional and interconnected vessels within the tissue. It is a complex process, involving interplay between resident cells, soluble factors and extracellular matrix components. Perhaps the most important mediator of angiogenesis is vascular endothelial growth factor (VEGF), recognised to be an endothelial cell-specific mitogen and chemotactic agent that plays a significant role in the physiology of the normal vasculature. Tissue engineered scaffolds have traditionally stimulated angiogenesis by the addition of single growth factors such as VEGF [50], however sequential addition of growth factors has improved on this approach [51]. Synthesis and release of angiogenic factors by cells within the construct could provide a better alternative to mimic the complex conditions required for angiogenesis. Secretome analysis of cells encapsulated within NapFF*ε*K*ε*K-OH hydrogels suggested that out of five bioactive factors (VEGF, fibroblast growth factor (FGF), epidermal growth factor (EGF), platelet-derived growth factor (PDGF) and insulin-like growth factor (IGF)) commonly utilised in tissue engineering [52], all but IGF were shown to increase in NapFF*ε*K*ε*K-OH hydrogel versus 2D culture. Interestingly, within the dental pulp IGF is associated with osteo/odontogenic differentiation [53,54] and levels may not therefore be expected to be so closely linked with angiogenesis or vascularisation.

## 5. Conclusions

In conclusion, this work has identified NapFF*ε*K*ε*K-OH hydrogels as biocompatible with cells of the dental pulp. Given that regenerative endodontics requires tissue regeneration following infection, and that bacterial elimination from infected canals is never complete, NapFF*ε*K*ε*K-OH showed biocompatibility with DPSCs and antibacterial activity against oral pathogens associated with endodontic infections, and therefore holds promise for translational use. Furthermore, the encapsulated cells within these antimicrobial hydrogels, produced angiogenic factors that could contribute to pulp revascularisation in vivo.

## Figures and Tables

**Table 1:** Stepwise formulation of a self-assembling pH-triggered 2% *w*/*v* NapFF*ε*K*ε*K peptide hydrogel.

Formulation Step	Constituent	Quantity
1	NapFFεKεK-OH Peptide	10 mg Preweighed
2	Deionized H2O	200 µL (in 50 µL aliquots)
3	1 M NaOH	50 µL (in 10 µL aliquots)
4	Deionized H_2_O	150 µL (in 50 µL aliquots)
5	0.5 M HCl	30 µL (in 10 µL aliquots)
6	Deionized H_2_O	50 µL
7	0.5 M HCl	to 500 µL (in 5 µL aliquots)

## Data Availability

The data presented in this study are available on request from the corresponding author.
